# Trends in High- and Low-Value Cardiovascular Diagnostic Testing in Fee-for-Service Medicare, 2000-2016

**DOI:** 10.1001/jamanetworkopen.2019.13070

**Published:** 2019-10-11

**Authors:** Vinay Kini, Timea Viragh, David Magid, Frederick A. Masoudi, Ali Moghtaderi, Bernard Black

**Affiliations:** 1 Department of Cardiology, University of Colorado Anschutz Medical Campus, Aurora; 2 Northwestern University School of Education and Social Policy, Evanston, Illinois; 3 George Washington University School of Public Health, Washington, DC; 4 Institute for Policy Research and Kellogg School of Management, Northwestern University Pritzker School of Law, Chicago, Illinois

## Abstract

**Importance:**

Owing to a rapid increase in rates of diagnostic cardiovascular testing in the 1990s and early 2000s, the Centers for Medicare & Medicaid Services implemented a series of payment changes intended to reduce overall spending on fee-for-service testing. Whether guideline-concordant testing has been subsequently affected is unknown to date.

**Objective:**

To determine whether changes in overall rates of use of diagnostic cardiovascular tests were associated with changes in high-value testing recommended by guidelines and low-value testing that is expected to provide minimal benefits.

**Design, Setting, and Participants:**

This retrospective cohort study assessed a national 5% random sample of Medicare fee-for-service beneficiaries aged 65 to 95 years from January 1, 1999, through December 31, 2016. Data were analyzed from February 15, 2018, through August 15, 2019.

**Exposures:**

Eligibility to receive high-value testing (assessment of left ventricular systolic function among patients hospitalized with acute myocardial infarction or heart failure) and low-value testing (stress testing before low-risk noncardiac surgery and routine stress testing within 2 years of coronary revascularization not associated with acute care visits).

**Main Outcomes and Measures:**

Age- and sex-adjusted annual rates of overall, high-value, and low-value diagnostic cardiovascular testing.

**Results:**

Mean (SD) age was similar over time (75.57 [7.32] years in 2000-2003; 74.82 [7.79] years in 2012-2016); the proportion of women slightly declined over time (63.23% in 2000 to 2003; 57.27% in 2012 to 2016). The rate of overall diagnostic cardiovascular testing per 1000 patient-years among the 5% sample of Medicare beneficiaries increased from 275 in 2000 to 359 in 2008 (*P* < .001) and then declined to 316 in 2016 (*P* < .001). High-value testing increased steadily over the entire study period for patients with acute myocardial infarction (85.7% to 89.5%; *P* < .001) and heart failure (72.6% to 80.1%; *P* < .001). Low-value testing among patients undergoing low-risk surgery increased from 2.4% in 2000 to 3.8% in 2008 (*P* < .001) but then declined to 2.5% in 2016 (*P* < .001). Low-value testing within 2 years of coronary revascularization slightly increased from 47.4% in 2000 to 49.2% in 2003 (*P* = .03) but then declined to 30.8% in 2014 (*P* < .001).

**Conclusions and Relevance:**

Rates of overall and low-value diagnostic cardiovascular testing appear to have declined considerably and rates of high-value testing have increased slightly. Payment changes intended to reduce spending on overall testing may not have adversely affected testing recommended by guidelines.

## Key Points

**Question:**

Are changes in annual rates of diagnostic cardiovascular tests associated with changes in rates of high- and low-value testing?

**Findings:**

In this cohort study of a 5% national sample of Medicare beneficiaries, annual rates of overall testing appeared to increase from 2000 to 2008 and then declined until 2016. Rates of low-value tests (preoperative stress testing and routine stress testing after coronary revascularization) appeared to have increased and then decreased, whereas rates of high-value tests (assessing left ventricular systolic function among patients hospitalized with acute myocardial infarction and heart failure) appeared to increase throughout the study period.

**Meaning:**

Payment changes intended to reduce spending on overall testing may not have adversely affected testing recommended by guidelines.

## Teaser

This cohort study assesses whether changes in overall rates of use of diagnostic cardiovascular tests were associated with changes in high-value testing recommended by guidelines and low-value testing that is expected to provide minimal benefits among a 5% national sample of Medicare beneficiaries between 2000 and 2006.

## Introduction

In the 1990s and early 2000s, the use of diagnostic cardiovascular testing such as stress tests and echocardiography among Medicare fee-for-service beneficiaries increased substantially.^1,2^ Coupled with significant geographic variation in the use and expense of these tests, potential overuse of testing was a concern.^3,4,5,6,7^ Beginning in 2004, the Centers for Medicare & Medicaid Services responded by implementing a series of reductions in the physician fees for inpatient and outpatient testing and the facility fees for office-based testing.^8^ In addition, prospective payments for hospitalizations based on diagnosis (such as the Medicare severity diagnosis related groups) removed facility fees for testing in hospitalized patients.^9,10^

In the face of these reimbursement changes, the overall rate of testing has modestly declined.^11,12,13^ However, to date whether these declines reflect use of high-value testing that is recommended by guidelines or low-value testing that is expected to provide minimal benefits is unknown. Prior studies suggest that payment changes intended to reduce spending on overall testing do not differentially reduce the use of high- and low-value medical procedures.^14,15^ If reductions in high- and low-value testing are observed, payment changes may have had the unintended consequence of reducing guideline-recommended testing. Accordingly, we examined use of (1) overall diagnostic cardiovascular testing, (2) high-value testing (assessment of left ventricular function among eligible patients hospitalized with acute myocardial infarction [AMI] or heart failure [HF]),^16,17,18^ and (3) low-value testing (stress testing before low-risk surgery and routine stress testing within 2 years of coronary revascularization with percutaneous coronary intervention [PCI] or coronary artery bypass graft [CABG] surgery) among Medicare fee-for-service beneficiaries from 2000 through 2016.^19,20,21^

## Methods

### Study Design, Data Source, and Study Population

We used a 5% random sample of Medicare fee-for-service beneficiaries aged 65 to 95 years from January 1, 1999, through December 31, 2016. The Medicare 5% random sample is drawn from the 100% Medicare administrative data file based on the Medicare health insurance claim number. An initial draw was made as of January 1, 1999, the start of our sample period. As beneficiaries leave the sample, generally through death or switch to Medicare Advantage, the sample is refreshed with additional beneficiaries, most of whom enter the sample at 65 years of age. Medicare beneficiaries younger than 65 years were not included because these beneficiaries are often recipients of Social Security disability or have end-stage renal disease and therefore may not be representative of the larger fee-for-service Medicare population. The institutional review board of George Washington University determined that this research was exempt from review and informed consent because it did not involve individually identifiable data. This study adheres to the reporting guidelines outlined in the Strengthening the Reporting of Observational Studies in Epidemiology (STROBE) reporting guideline.

The data set includes beneficiary demographic and enrollment data. We used codes from *International Classification of Diseases, Ninth Revision*, *International Statistical Classification of Diseases and Related Health Problems, Tenth Revision*, and *Current Procedural Terminology* (eTable 1 and eTable 2 in the Supplement) to identify high- and low-value tests. Each patient’s first enrollment year was used to measure comorbidities and prior procedures that could affect whether tests fall within the high- and low-value categories. Diagnostic tests performed during each patient’s first enrollment year were excluded.

### High- and Low-Value Cardiovascular Tests

We studied the following diagnostic cardiovascular tests: echocardiography (transthoracic and transesophageal), stress echocardiography, stress electrocardiography, nuclear single-photon emission computed tomography (SPECT), left heart catheterization with left ventriculography, nuclear positron emission tomography, coronary computed tomography with angiography, and cardiac magnetic resonance imaging. We then identified high- and low-value tests as follows.

#### High-Value Testing Among Patients With AMI

**Figure 1.**
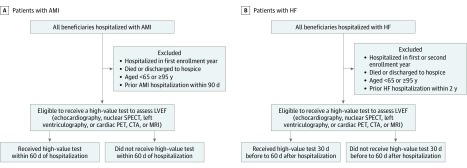
Flow Diagrams for Creation of High-Value Testing Cohorts AMI indicates acute myocardial infarction; CTA, computed tomography angiography; HF, heart failure; LVEF, left ventricular ejection fraction; MRI, magnetic resonance imaging; PET, positron emission tomography; and SPECT, single-photon emission computed tomography.

Using methods previously validated by Miller et al,^22^ we identified all patients discharged with a primary diagnosis of AMI and excluded those in whom left ventricular ejection fraction (LVEF) assessment may not have been indicated according to clinical practice guidelines.^16,17,18^ This group included patients who died during hospitalization or were discharged to hospice and patients readmitted with AMI within 90 days of a prior hospitalization, because readmission may have been related to the initial event and would not necessarily require another LVEF assessment (Figure 1A). If the same patient had another AMI hospitalization 90 days after a prior AMI hospitalization, they were considered eligible for high-value testing again. We identified whether a test to assess LVEF (echocardiography, nuclear SPECT, left heart catheterization with left ventriculography, or cardiac positron emission tomography, computed tomography with angiography, or magnetic resonance imaging) was performed within 60 days after hospitalization for AMI. We performed a sensitivity analysis in which we examined trends separately for patients with and without ST-segment elevation because some patients with a discharge diagnosis of non–ST-segment elevation AMI may not require LVEF assessment (eg, miscoding of non-AMI elevation of troponin levels).^23^

#### High-Value Testing Among Patients With HF

Using methods similar to those previously validated by Curtis et al^24^ and Farmer et al,^25^ we identified all patients discharged with a primary diagnosis of HF and excluded those in whom LVEF assessment may not have been indicated according to clinical practice guidelines. This group included patients who died during their hospitalization or were discharged to hospice and those with HF hospitalizations within 2 years of a prior hospitalization, because LVEF assessment may not be necessary in patients with recent prior hospitalizations and in whom a clear HF precipitant was noted (Figure 1B). If the same patient had another HF hospitalization 2 years after a prior HF hospitalization, they were considered eligible for high-value testing again. We identified whether a test to assess LVEF (echocardiography, nuclear SPECT, left heart catheterization with left ventriculography, or cardiac positron emission tomography, computed tomography with angiography, or magnetic resonance imaging) was performed from 30 days before (to capture tests that may have prompted hospitalization) to 60 days after hospitalization. For sensitivity analyses, we examined trends in high-value testing separately for patients with incident HF (ie, no prior HF diagnosis), and varied the periods allowed for testing from 60 days before to 90 days after hospitalization.

#### Low-Value Testing Among Patients Undergoing Low-Risk Surgery

**Figure 2.**
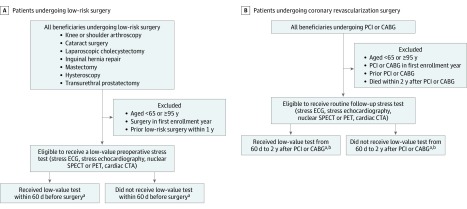
Flow Diagrams for Creation of Low-Value Testing Cohorts CABG indicates coronary artery bypass graft surgery; CTA, computed tomography angiography; ECG, electrocardiography; PCI, percutaneous coronary intervention; PET, positron emission tomography; and SPECT, single-photon emission computed tomography. ^a^Tests related to acute care (emergency department visits or hospital admissions) were not considered low value. ^b^Tests that were followed by repeated coronary revascularization were not considered low value.

Among all patients undergoing low-risk surgery (knee or shoulder arthroscopy, cataract surgery, laparoscopic cholecystectomy, inguinal hernia repair, mastectomy, hysteroscopy, or transurethral prostatectomy), we identified patients for whom cardiac testing was likely to be low value based on appropriate use criteria and Choosing Wisely (Figure 2A).^19,20,21^ We used methods previously validated by Kerr et al^26^ and Schwartz et al.^27^ We treated as low-value tests any stress test performed in the 60 days before the low-risk surgery. We excluded operations that occurred within 1 year after a prior surgery. If the same patient had another low-risk surgery 1 year after a prior surgery, they were considered eligible for low-value testing again. To isolate routine preoperative stress tests from those that may have been performed for other reasons, we excluded tests within 3 days before or after an emergency department visit or within 30 days of another hospitalization.

#### Low-Value Testing Among Patients With PCI or CABG

For patients who underwent PCI or CABG, we identified those for whom stress testing was likely to be low value based on appropriate use criteria and Choosing Wisely (Figure 2B).^19,20,21^ We used methods similar to those of Shah et al^28^ and Bradley et al.^29^ We identified all patients who underwent an initial index PCI or CABG. We excluded subsequent PCI and CABG procedures in the same patient (ie, patients were eligible for low-value testing only once). We identified whether patients received a cardiac stress test from 60 days to 2 years after the index procedure. We excluded stress tests within 60 days of the index procedure because these tests may have been performed to guide staged revascularization (ie, PCI of intermediate-severity stenosis identified during the index PCI procedure). To isolate routine, low-value tests from those that may have been performed for other reasons, we excluded tests that occurred within 3 days before or after an emergency department visit that occurred within 30 days of a hospitalization or that were followed by a subsequent PCI or CABG procedure within 30 days. For sensitivity analyses, we varied the interval allowed for testing to 1 year and 6 months after the index procedure, and we counted as low-value (ie, did not exclude) tests that were followed by a subsequent PCI or CABG procedure within 30 days.

### Statistical Analysis

Data were analyzed from February 15, 2018, through August 15, 2019. The primary measures were the annual rates of overall diagnostic testing and each high- or low-value test in the populations eligible for these tests. Covariates included age and sex. Race/ethnicity and the 17 elements of the Charlson comorbidity index were extracted for descriptive analysis.^30^ Charlson comorbidity indices were assessed on a rolling basis using the 4 calendar quarters preceding the quarter in which the patient was eligible for the high- or low-value test. Differences in patient characteristics over time were assessed using χ^2^ tests for categorical variables and unpaired *t* tests for continuous variables. We measured overall testing rates per 1000 patient-years for Medicare beneficiaries with at least 1 month of Medicare fee-for-service coverage in the year of testing. For high- and low-value testing, the denominator was the number of patients eligible to receive a high- or low-value test for each clinical scenario; the numerator consisted of the number of patients who actually received the test. Patients who were eligible for more than 1 high- or low-value test in a given year were counted each time in the denominator; patients who received more than 1 high- or low-value test in a given year were counted each time in the numerator. We allowed for overlap between time periods of patients eligible to receive high- and low-value testing in different scenarios (eg, a patient undergoing low-risk surgery and then hospitalized for AMI could have been counted in both the low- and high-value testing cohorts). Annual testing rates were adjusted for age and sex using the indirect adjustment method, with the mean overall testing rate during the sample period as the standard rate.^31^ We used linear regression models with 2-sided *P* values to assess the association between rates of testing and time (the key independent variable), with *P* < .05 indicating significance. For trends that visually appeared to be nonlinear, we used Chow tests to confirm breakpoints in linearity and separate linear regressions to evaluate for trends before and after break points. Stata software, version 13.1 (StataCorp, LLC), was used for statistical analysis.

## Results

### Patient Characteristics

Comparison of the characteristics of patients in our sample revealed slight differences over time (eTable 3 in the Supplement). Mean (SD) age was similar over time (75.57 [7.32] years in 2000-2003; 74.82 [7.79] years in 2012-2016); the proportion of women slightly declined over time (63.23% in 2000 to 2003; 57.27% in 2012 to 2016). The prevalence of most comorbid conditions increased over time. Comparison of the characteristics of patients eligible to receive high- and low-value testing revealed similar differences over time (eTable 4 and eTable 5 in the Supplement). The proportion of patients eligible for more than 1 high- or low-value test throughout the study period was 5.9%.

### Temporal Trends in Overall Diagnostic Cardiovascular Testing

**Figure 3.**
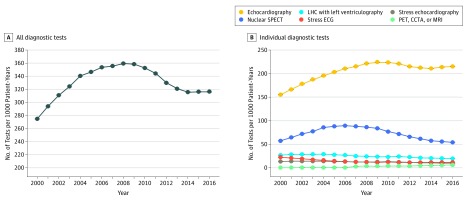
Annual Rates of Diagnostic Cardiovascular Tests All testing rates are adjusted for age and sex. CCTA indicates cardiac computed tomography angiography; ECG, electrocardiography; LHC, left heart catheterization; MRI, magnetic resonance imaging; PET, positron emission tomography; and SPECT, single-photon emission computed tomography.

The overall diagnostic testing rate per 1000 person-years increased from 275 in 2000 to 359 in 2008 (*P* < .001) and then steadily declined to 316 in 2016 (*P* < .001) (Figure 3A). The percentage decline from 2008 to 2016 was 12%. Echocardiography and SPECT, the 2 most common diagnostic tests, exhibited similar temporal trends (Figure 3B). The rate of echocardiography per 1000 person-years increased from 141 in 1999 to 227 in 2009 (*P* < .001), and then declined to 210 in 2016 (*P* < .001). The percentage decline from 2009 to 2016 was 7%. The rate of SPECT per 1000 person-years increased from 48 in 1999 to 89 in 2006 (*P* < .001), and then declined to 51 in 2016 (*P* < .001). The percentage decline in SPECT rates from 2006 to 2016 was 43%. The ratio of high- and low-value tests we studied to the total number of tests performed each year ranged from 4.5% to 8.5% annually throughout the study period.

### Temporal Trends in High-Value Testing

**Figure 4.**
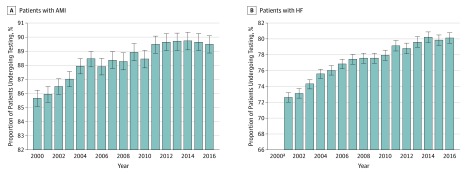
Annual Rates of High-Value Testing All testing rates are adjusted for age and sex. Error bars show 95% CIs. AMI indicates acute myocardial infarction; HF, heart failure. ^a^Annual rates begin in 2001 to allow for 2-year look-back period.

For the AMI cohort, the number of eligible patients declined from 14 456 in 2000 to 9824 in 2016. The decline in the population eligible for testing was similar to declines in the overall population (eFigure 1A in the Supplement). The proportion of eligible patients who received high-value testing increased from 85.7% in 2000 to 89.5% in 2016 (*P* < .001) (Figure 4A). A sensitivity analysis showed that this increase was largely owing to increases in rates of testing among patients with non–ST-segment elevation AMI; testing rates among patients with ST-segment elevation AMI were high throughout the study period (range, 89% to 92%) (eFigure 2 in the Supplement). For the HF cohort, the number of eligible patients declined at a rate similar to that of the overall population from 21 382 in 2000 to 14 339 in 2016 (eFigure 1B in the Supplement). The proportion of eligible patients who received high-value testing steadily increased from 72.6% in 2000 to 80.1% in 2016 (*P* < .001) (Figure 4B). A sensitivity analysis among patients hospitalized with incident HF showed similar increases in high-value testing over time (eFigure 3 in the Supplement), as did a sensitivity analysis in which we varied the periods allowed for testing (eFigure 4 in the Supplement).

### Temporal Trends in Low-Value Testing

**Figure 5.**
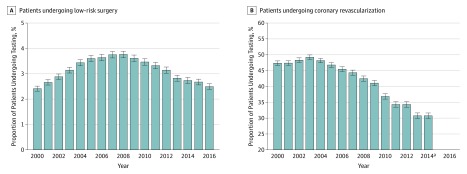
Annual Rates of Low-Value Testing All testing rates are adjusted for age and sex. Error bars show 95% CIs. ^a^Annual rates end in 2014 to allow for 2-year look-forward period.

For the low-risk surgery cohort, the number of eligible patients declined from 93 901 in 2000 to 79 405 in 2016 (eFigure 1C in the Supplement). The proportion of eligible patients who received low-value testing before low-risk surgery increased from 2.4% in 2000 to 3.8% in 2008 (*P* < .001) and then declined to 2.5% in 2016 (*P* < .001) (Figure 5A). The decline in rate from 2008 to 2016 was 34%, similar in magnitude to the increase from 2000 to 2008. For the coronary revascularization cohort, the number of eligible patients declined from 19 230 in 2000 to 10 810 in 2014 (eFigure 1D in the Supplement). The proportion of eligible patients who received low-value testing within 2 years after PCI or CABG slightly increased from 47.4% in 2000 to 49.2% in 2003 (*P* = .03), then progressively declined to 30.8% in 2014 (*P* < .001) (Figure 5B). Sensitivity analyses varying the period allowed for testing and counting tests followed by subsequent PCI or CABG as low value showed similar trends over time (eFigure 5 and eFigure 6 in the Supplement). Unadjusted annual rates of each high- and low-value test were similar to rates adjusted for age and sex (eFigure 7 in the Supplement).

## Discussion

### Summary of Findings and Extension of Prior Research

In this study of trends in the use of cardiovascular diagnostic testing among fee-for-service Medicare beneficiaries from 1999 to 2016, we found that the overall rate of testing increased from 1999 to 2008 and then steadily declined through 2016. High-value testing among eligible patients hospitalized for AMI and HF steadily increased for both cohorts throughout the study period, approaching 90% for the AMI cohort and 80% for the HF cohort. Low-value testing before low-risk surgery was performed infrequently but increased from 2000 to 2008 and declined thereafter, returning by 2015 to the 2000 level. Low-value testing after PCI or CABG slightly increased from 2000 to nearly 50% in 2003, but then steadily declined to approximately 30%.

Prior studies^1,2,11,12,13^ have found similar declines in diagnostic testing rates since 2008. Changes in reimbursement for testing constitute one of several possible reasons that rates of testing declined, raising the possibility that high- and low-value testing might have been affected. Furthermore, recent meta-analyses^32,33^ have found no significant change in rates of appropriateness of testing since 2008, raising additional concerns that the observed declines in overall testing may reflect declines in use of high-value testing. We find that along with increases in the overall rate of testing in the early 2000s, concurrent increases occurred in high- and low-value testing. During the period from 2008 forward in which overall testing rates fell, continued increases in high-value testing for patients with AMI and HF occurred, as well as declines in low-value testing rates before low-risk surgery (reversing the prior trend) and after revascularization (continuing a trend that began in 2003). The fractional declines in low-value testing rates are substantially larger than the decline in overall testing rates. Our findings suggest that during a period of Medicare reimbursement changes intended to reduce spending on overall testing, rates of low-value testing declined considerably while guideline-concordant testing among patients with AMI and HF was not adversely affected.

### Factors Potentially Associated With Use of Testing

Reimbursement reductions have previously been shown to reduce rates of use of health care services.^14,15^ Beginning in 2004, the Centers for Medicare & Medicaid Services implemented a multiyear cut in reimbursement for the physician component of testing among inpatients and outpatients and the facility component of office-based echocardiography and stress testing.^8^ These reimbursement cuts were implemented during a series of years and at different points for different testing modalities. Inpatient diagnostic testing for patients hospitalized for AMI and HF is part of a prospective payment bundle (the Medicare severity diagnosis related group), and there is no facility fee-for-service incentive to perform testing among these patients. Concerns have been raised that these policies may lead to declines in high-value diagnostic tests that are recommended by guidelines. For example, a reduction in reimbursement for dual-energy x-ray absorptiometry scans in Canada led to underuse of testing among patients likely to benefit from receiving the test.^34^ Other studies^14,15,35^ have shown that prospective payment bundles may lead to reduced use of health care services and worse patient outcomes. Our study shows that rates of high-value testing among eligible patients with AMI and HF did not decline in the face of these reimbursement changes. Still, reimbursement changes differed during this period for hospital-based and office-based outpatient testing; further research should be performed to examine differences in high- and low-value testing across these settings.

In response to concerns about overuse of testing, cardiovascular professional societies disseminated clinical practice guidelines to improve the use of testing. The American College of Cardiology published methods for development of appropriate use criteria for cardiac imaging in 2005,^36^ with subsequent publications and revisions for echocardiography and stress testing underway 2007 to 2012.^19,20^ Clinical practice guidelines for AMI and perioperative testing have been continually updated every few years since the early 2000s. The American Board of Internal Medicine’s Choosing Wisely campaign was published in 2012 and included the 2 low-value tests we examined in this study.^21^ Prior studies have found conflicting evidence on the influence of guideline dissemination on practice patterns.^37,38^ Although these efforts may have contributed to observed declines in the rate of low-value testing and increases in high-value testing, we cannot establish a causal connection in our study. The rate of low-value testing before low-risk surgery peaked in 2008 (around the time of publication of the appropriate use criteria) and subsequently declined, with no apparent change in trend after the publication of Choosing Wisely in 2012. The rate of low-value testing after PCI or CABG began to decline in 2003, also with no apparent change in trend after publication of the appropriate use criteria and Choosing Wisely. Other changes during the study period such as implementation of public reporting of hospital outcomes, vertical integration of cardiology practices into health systems, value-based physician incentives, or other clinical practice changes not addressed in guideline documents could also have contributed to the observed changes in testing rates.

### Limitations

We studied trends in overall diagnostic cardiac testing and in high-value and low-value testing in specific scenarios that can be assessed using administrative claims data. Trends for other high- and low-value tests, as well as tests of uncertain appropriateness, may differ and were not assessed in this study because the details required to categorize these tests were unavailable in our administrative data. The ratio of high- and low-value tests we studied to the total number of tests performed each year ranged from 4.5% to 8.5% annually throughout the study period. We studied trends in high- and low-value testing over a period that included multiyear reductions in Centers for Medicare & Medicaid payments for testing and publications and revisions of clinical practice guidelines. Causal connections between changes in high- and low-value testing and payment reductions or guideline publication cannot be established by our study. We used a sample of Medicare fee-for-service beneficiaries, which may not be generalizable to other populations such as beneficiaries enrolled in Medicare Advantage programs. There were significant declines in our denominator population eligible for high- and low-value testing, but similar declines were observed in the overall rate of hospitalizations for AMI and HF, low-risk operations, and PCI or CABG in the overall Medicare 5% sample (eFigure 1 in the Supplement). These observations suggest that the declines reflect trends in the overall population. Unmeasured confounders such as reduced access to AMI or HF care for marginalized populations over time could also have affected testing rates. Reporting of comorbid conditions increased in frequency over the study period, which could have had the effect of increasing use of testing among patients hospitalized for AMI or HF, because increasing complexity of patients may prompt the use of more tests. However, reported comorbid conditions also appeared to increase among patients undergoing low-risk surgery and stress testing after PCI or CABG, and increases in testing were not observed in these patients.

## Conclusions

Rates of overall and low-value diagnostic cardiovascular testing appear to have declined considerably, and rates of high-value testing appear to have increased slightly. Payment changes intended to reduce spending on overall testing may not have adversely affected testing recommended by guidelines.

## Notes

Supplement. **eTable 1.** *CPT* Codes Used to Identify Diagnostic Cardiovascular Tests and Low-Risk Operations **eTable 2.** *ICD-9* and *ICD-10* Codes Used to Identify Hospitalizations for Acute Myocardial Infarction (AMI) and Heart Failure (HF) **eTable 3.** Characteristics of Patients in the Medicare 5% Sample **eTable 4.** Characteristics of Patients Eligible to Receive High-Value Testing **eTable 5.** Characteristics of Patients Eligible to Receive Low-Value Testing **eFigure 1.** Annual Counts of Services per Beneficiary-Year Among the Overall Population (5% Medicare Fee-for-Service Sample) and the Study Cohorts Eligible for High- and Low-Value Testing **eFigure 2.** AMI Sensitivity Analysis **eFigure 3.** Heart Failure Sensitivity Analysis: Annual Rate of High-Value Testing for Patients Hospitalized With Incident Heart Failure **eFigure 4.** Heart Failure Sensitivity Analysis: Trends in High-Value Testing Among HF Patients, Varying the Time Period for Measuring Testing Rates **eFigure 5.** Coronary Revascularization Sensitivity Analysis: Trends in Low-Value Testing Within 2 Years, 1 Year, and 6 Months of Coronary Revascularization **eFigure 6.** Coronary Revascularization Sensitivity Analysis: Trends in Low-Value Testing Within 2 Years Without Excluding Tests That Were Followed by Subsequent PCI/CABG Within 30 Days **eFigure 7.** Unadjusted Annual Rates of High- and Low-Value Testing
