# Supplementary material for: Trends in High- and Low-Value Cardiovascular Diagnostic Testing in Fee-for-Service Medicare, 2000-2016
Source: JAMA Netw Open. 2019 Oct 11;2(10):e1913070. doi: 10.1001/jamanetworkopen.2019.13070 (PMC6804029; doi:10.1001/jamanetworkopen.2019.13070)
Supplement: Supplement. — eTable 1. CPT Codes Used to Identify Diagnostic Cardiovascular Tests and Low-Risk Operations eTable 2. ICD-9 and ICD-10 Codes Used to Identify Hospitalizations for Acute Myocardial Infarction (AMI) and Heart Failure (HF) eTable 3. Characteristics of Patients in the Medicare 5% Sample eTable 4. Characteristics of Patients Eligible to Receive High-Value Testing eTable 5. Characteristics of Patients Eligible to Receive Low-Value Testing eFigure 1. Annual Counts of Services per Beneficiary-Year Among the Overall Population (5% Medicare Fee-for-Service Sample) and the Study Cohorts Eligible for High- and Low-Value Testing eFigure 2. AMI Sensitivity Analysis eFigure 3. Heart Failure Sensitivity Analysis: Annual Rate of High-Value Testing for Patients Hospitalized With Incident Heart Failure eFigure 4. Heart Failure Sensitivity Analysis: Trends in High-Value Testing Among HF Patients, Varying the Time Period for Measuring Testing Rates eFigure 5. Coronary Revascularization Sensitivity Analysis: Trends in Low-Value Testing Within 2 Years, 1 Year, and 6 Months of Coronary Revascularization eFigure 6. Coronary Revascularization Sensitivity Analysis: Trends in Low-Value Testing Within 2 Years Without Excluding Tests That Were Followed by Subsequent PCI/CABG Within 30 Days eFigure 7. Unadjusted Annual Rates of High- and Low-Value Testing [file jamanetwopen-2-e1913070-s001.pdf]

Supplementary Online Content

Kini V, Viragh T, Magid D, Masoudi FA, Moghtaderi A, Black B. Trends in high- and low-value cardiovascular diagnostic testing in fee-for-service Medicare, 2000-2016. *JAMA Netw Open.* 2019;2(10):e1913070. Published online October 11, 2019. doi:10.1001/jamanetworkopen.2019.13070

eTable 1. *CPT* Codes Used to Identify Diagnostic Cardiovascular Tests and Low-Risk Operations

eTable 2. *ICD-9* and *ICD-10* Codes Used to Identify Hospitalizations for Acute Myocardial Infarction (AMI) and Heart Failure (HF)

eTable 3. Characteristics of Patients in the Medicare 5% Sample

eTable 4. Characteristics of Patients Eligible to Receive High-Value Testing

eTable 5. Characteristics of Patients Eligible to Receive Low-Value Testing

eFigure 1. Annual Counts of Services per Beneficiary-Year Among the Overall Population (5% Medicare Fee-for-Service Sample) and the Study Cohorts Eligible for High- and Low-Value Testing

eFigure 2. AMI Sensitivity Analysis

eFigure 3. Heart Failure Sensitivity Analysis: Annual Rate of High-Value Testing for Patients Hospitalized With Incident Heart Failure

eFigure 4. Heart Failure Sensitivity Analysis: Trends in High-Value Testing Among HF Patients, Varying the Time Period for Measuring Testing Rates

eFigure 5. Coronary Revascularization Sensitivity Analysis: Trends in Low-Value Testing Within 2 Years, 1 Year, and 6 Months of Coronary Revascularization

eFigure 6. Coronary Revascularization Sensitivity Analysis: Trends in Low-Value Testing Within 2 Years Without Excluding Tests That Were Followed by Subsequent PCI/CABG Within 30 Days

eFigure 7. Unadjusted Annual Rates of High- and Low-Value Testing

This supplementary material has been provided by the authors to give readers additional information about their work.

© 2019 Kini V et al. *JAMA Network Open.*

eTable 1. *CPT* Codes Used to Identify Diagnostic Cardiovascular Tests and Low-Risk Operations

Procedure	Main codes	Add-on codes	Notes
**Echocardiography:**			
Transesophageal Echo (TEE)	93312, 93313, 93314, 93315, 93316, 93317, 93318, C8925, C8926, C8927, G9157	93320, 93321, 93325, 93352, A9700, C1579	Code G9157 is used from 2013-2014. Codes C8925, C8926 and C8927 are used from 2008-2014. Code 93318 is used from 2005-2015. All other codes are used from 2005-2016.
Transthoracic Echo (TTE)	93303, 93304, 93306, 93307, 93308, C8921, C8922, C8923, C8924, C8929	-	All codes are used from 2005-2016.
**Stress tests:**			
Stress Electrocardiogram (ECG)	93015, 93016, 93017, 93018	-	All codes are used from 2005-2016.
Single-photon emission computed tomography (SPECT)	78451, 78452, 78464, 78465, 78468, 78469	78478, 78480	Codes 78451 and 78452 are used from 2010-2016. Codes 78464, 78465, 78478 and 78480 are used from 2005-2009. All other codes are used from 2005-2016.
Stress echocardiogram (Stress Echo)	93350, 93351, C8928, C8930, G8961, G8962	93015, 93016, 93017, 93018, 93320, 93321, 93325, 93352, A9700, C1759	Code 93015 is used from 2005-2008. Codes 93351 and C8930 are used from 2009-2016. Code C8928 is used from 2008-2016. Codes G8961 and G8962 are used from 2013-2016. All other codes are used from 2005-2016.
Cardiac computed tomography angiography (CCTA)	75571, 75572, 75573, 75574, 76497, S8093, 0144T, 0145T, 0146T, 0147T, 0148T, 0149T, 0150T	-	Code 76497 is used for 2003-2015. Code S8093 is used for 2004-2005, codes 0144T-0150T are used for 2007-2009. Codes 75571-75574 are used for 2010-2016.
Nuclear Positron Emission Tomography (PET)	78459, 78491, 78492	-	All codes are used from 2005-2016.
Stress Cardiac MRI	75559, 75563	-	All codes are used from 2008-2016.
**Left heart catheterization with left ventriculography**	93543, 93451, 93452, 93453, 93458, 93459, 93460, 93461, 93563, 93564, 93565, 93566, 93567, 93568	-	Code 93543 is used from 1999-2010. All other codes are used from 2011-2016.

© 2019 Kini V et al. *JAMA Network Open.*

**Low-risk surgeries:**			
Knee arthroscopy	CPT 29866-29868, 29870, 29873-29877, 29879-29889		
Shoulder arthroscopy	CPT 29805-29807, 29819-29828		
**Procedure**	**Main codes**	**Add-on codes**	**Notes**
Cataract surgery	CPT 66982, 66984		
Laparoscopic cholecystectomy	CPT 47562, 47563		
Inguinal hernia repair	CPT 49650, 49651		
Mastectomy	For 2000-2006: CPT 19180, 19182, 19200, 19220, 19240<br>For 2007-2016: CPT 19303, 19304, 19305, 19306, 19307		
Hysteroscopy	CPT 58558		
Transurethral Prostatectomy	CPT 52601		

© 2019 Kini V et al. *JAMA Network Open*.

eTable 2. ***ICD-9* and *ICD-10* Codes Used to Identify Hospitalizations for Acute Myocardial Infarction (AMI) and Heart Failure (HF)**

**Diagnosis**	**ICD-9**	**ICD-10**
Heart Failure (HF)	402.XX, 404.XX, 428.XX	I09.81, I11.0, I13.0, I13.2, I50.XX
Acute Myocardial Infarction (AMI)	410.0-410.9	
ST-segment elevation	410.0-410.6, 410.8	I21.0-I21.3
Non-ST-segment elevation	410.7, 410.9	I21.4

© 2019 Kini V et al. *JAMA Network Open.*

eTable 3. Characteristics of Patients in the Medicare 5% Sample

	2000-2003	2004-2007	2008-2011	2012-2016
**Average patients per year**	1,736,058	1,727,227	1,653,372	1,722,524
**Age** (mean +/- SD)	75.57 +/- 7.32	75.66 +/- 7.50	75.49 +/- 7.74	74.82 +/- 7.79
**Female*** (n, %)	1,097,459 (63.23)	1,061,616 (61.45)	983,283 (59.47)	986,365 (57.27)
**Race*** (n, %)				
White	1,512,810 (87.14)	1,498,064 (86.73)	1,422,446 (86.04)	1,454,804 (84.47)
Black	136,685 (7.87)	134,179 (7.76)	127,541 (7.71)	135,041 (7.84)
Hispanic	33,732 (1.94)	31,774 (1.84)	30,903 (1.87)	32,024 (1.86)
Asian	25,406 (1.46)	29,434 (1.71)	33,187 (2.01)	37,647 (2.18)
Other/Unknown	27,426 (1.58)	33,776 (1.96)	39,294 (2.38)	63,008 (3.65)
**Comorbidities*** (n, %)				
Diabetes Without Complication	374,291 (23.04)	430,846 (26.51)	444,726 (29.01)	448,840 (28.42)
Chronic Pulmonary Disease	390,462 (24.05)	409,300 (25.16)	383,981 (25.05)	383,055 (24.25)
Peripheral Vascular Disease	269,307 (16.58)	308,757 (19.00)	315,368 (20.57)	312,498 (19.78)
Cerebrovascular Disease	289,870 (17.85)	314,663 (19.35)	306,577 (20.00)	284,877 (18.05)
Malignancy	234,358 (14.44)	240,734 (14.80)	231,500 (15.10)	229,070 (14.50)
Congestive Heart Failure	283,193 (17.45)	280,106 (17.21)	248,703 (16.22)	225,015 (14.25)
Renal Disease	74,579 (4.58)	116,023 (7.17)	171,525 (11.19)	220,574 (13.96)
Diabetes With Complication	98,890 (6.08)	122,882 (7.56)	140,473 (9.16)	171,936 (10.86)
Myocardial Infarction	112,068 (6.90)	111,744 (6.86)	102,421 (6.68)	102,655 (6.50)
Dementia	96,033 (5.92)	102,738 (6.32)	100,453 (6.55)	99,269 (6.28)
Mild Liver Disease	61,059 (3.76)	76,556 (4.71)	80,459 (5.25)	89,915 (5.69)
Rheumatic Disease	77,652 (4.78)	78,180 (4.80)	75,013 (4.89)	75,079 (4.75)
Peptic Ulcer Disease	61,994 (3.83)	49,292 (3.02)	38,150 (2.49)	35,690 (2.26)
Metastatic Solid Tumor	39,152 (2.41)	37,372 (2.30)	33,396 (2.18)	33,551 (2.12)
Hemiplegia/Paraplegia	32,340 (1.99)	27,582 (1.69)	24,377 (1.59)	24,082 (1.52)
Moderate/Severe Liver Disease	4,550 (0.28)	5,033 (0.31)	5,689 (0.37)	6,816 (0.43)
AIDS/HIV	766 (0.05)	1,021 (0.06)	1,210 (0.08)	1,709 (0.11)

*Counts and percentages are expressed per year, averaged over each time interval

© 2019 Kini V et al. *JAMA Network Open*.

eTable 4. Characteristics of Patients Eligible to Receive High-Value Testing

	Acute Myocardial Infarction				Heart Failure			
	2000-2003	2004-2007	2008-2011	2012-2016	2001-2003	2004-2007	2008-2011	2012-2016
Number of patients (mean across years)	14,684	12,446	10,186	9,526	21,233	19,295	15,785	14,018
**Age** (mean, SD)	78.43<br>7.27	78.72<br>7.42	78.69<br>7.74	78.10<br>7.88	80.19<br>6.98	80.75<br>7.07	81.24<br>7.22	81.14<br>7.50
**Female*** (n,%)	8,263<br>56.27	6,866<br>55.15	5,368<br>52.67	4,709<br>49.43	13,584<br>63.98	11,970<br>62.01	9,595<br>60.77	8,078<br>57.64
**Race*** (n,%)								
White	13,025<br>88.70	10,926<br>87.79	8,938<br>87.73	8,190<br>85.97	18,152<br>85.49	16,554<br>85.80	13,495<br>85.49	11,864<br>84.63
Black	1,025<br>6.98	930<br>7.48	772<br>7.60	790<br>8.29	2,256<br>10.63	1,993<br>10.32	1,614<br>10.22	1,486<br>10.60
Hispanic	249<br>1.69	215<br>1.73	173<br>1.70	164<br>1.72	424<br>2.00	354<br>1.83	297<br>1.88	237<br>1.69
Asian	98<br>0.67	126<br>1.01	113<br>1.11	137<br>1.44	164<br>0.77	164<br>0.85	172<br>1.09	192<br>1.37
Other / Unknown	288<br>1.96	249<br>1.99	191<br>1.87	246<br>2.58	237<br>1.12	232<br>1.20	208<br>1.32	239<br>1.70
**Comorbid Conditions*** (n,%)								
Myocardial Infarction	2,537<br>17.85	2,108<br>17.26	1,792<br>18.13	1,787<br>19.30	3,519<br>17.16	3,257<br>17.26	2,708<br>17.84	2,656<br>19.59
Congestive Heart Failure	4,475<br>31.48	3,740<br>30.66	3,054<br>30.88	2,643<br>28.54	11,676<br>56.97	10,941<br>58.03	9,063<br>59.70	8,033<br>59.24
Peripheral Vascular Disease	3,327<br>23.40	3,248<br>26.71	2,901<br>29.36	2,769<br>29.90	5,886<br>28.70	6,108<br>32.48	5,486<br>36.16	5,261<br>38.81
Cerebrovascular Disease	3,209<br>22.57	2,902<br>23.83	2,516<br>25.46	2,284<br>24.68	5,462<br>26.64	5,290<br>28.07	4,614<br>30.39	4,049<br>29.87
Dementia	830<br>5.83	822<br>6.75	703<br>7.11	603<br>6.50	1,436<br>7.00	1,468<br>7.81	1,336<br>8.80	1,233<br>9.10
Chronic Pulmonary Disease	3,930<br>27.64	3,615<br>29.70	3,071<br>31.08	2,879<br>31.10	7,737<br>37.74	7,648<br>40.60	6,653<br>43.87	6,413<br>47.30
Rheumatic Disease	650<br>4.57	594<br>4.89	505<br>5.10	485<br>5.24	1,093<br>5.33	1,040<br>5.51	902<br>5.95	883<br>6.51
Peptic Ulcer Disease	567<br>3.99	363<br>2.94	258<br>2.61	248<br>2.67	984<br>4.80	723<br>3.80	540<br>3.55	478<br>3.52
Mild Liver Disease	444<br>3.12	437<br>3.60	423<br>4.28	441<br>4.75	874<br>4.26	906<br>4.82	832<br>5.49	854<br>6.30
Diabetes Without Complication	5,122<br>36.03	4,655<br>38.23	4,077<br>41.28	3,884<br>41.96	8,563<br>41.76	8,395<br>44.56	7,192<br>47.40	6,615<br>48.79
Diabetes With Complication	1,665<br>11.71	1,679<br>13.83	1,664<br>16.86	1,886<br>20.36	3,116<br>15.19	3,251<br>17.29	3,139<br>20.71	3,460<br>25.53
Hemiplegia / Paraplegia	317<br>2.23	232<br>1.89	184<br>1.86	165<br>1.78	571<br>2.79	429<br>2.26	348<br>2.29	303<br>2.24
Renal Disease	1,367<br>9.60	1,688<br>14.04	2,055<br>20.87	2,339<br>25.27	3,036<br>14.79	3,951<br>21.21	4,795<br>31.67	5,378<br>39.66
Malignancy	1,830<br>12.87	1,645<br>13.51	1,378<br>13.93	1,296<br>14.00	2,939<br>14.33	2,864<br>15.21	2,482<br>16.36	2,278<br>16.80
Moderate / Severe Liver Disease	35<br>0.24	29<br>0.23	30<br>0.31	37<br>0.39	77<br>0.37	79<br>0.42	88<br>0.58	94<br>0.69

© 2019 Kini V et al. *JAMA Network Open.*

	Acute Myocardial Infarction				Heart Failure			
	2000-2003	2004-2007	2008-2011	2012-2016	2001-2003	2004-2007	2008-2011	2012-2016
Metastatic Solid Tumor	244<br>1.71	224<br>1.84	183<br>1.85	169<br>1.83	468<br>2.28	442<br>2.34	350<br>2.30	337<br>2.49
AIDS/HIV	8<br>0.06	6<br>0.05	10<br>0.10	10<br>0.11	14<br>0.07	14<br>0.07	15<br>0.10	13<br>0.10

* Counts and percentages are expressed per year, averaged over each time interval

© 2019 Kini V et al. *JAMA Network Open*.

eTable 5. Characteristics of Patients Eligible to Receive Low-Value Testing

	Low-Risk Surgery				PCI/CABG			
	2000-2003	2004-2007	2008-2011	2012-2016	2000-2003	2004-2007	2008-201	2012-2016
Number of patients (mean across years)	90,828	88,986	80,274	76,860	18,779	16,927	12,713	10,819
**Age** (mean, SD)	76.61<br>6.32	76.39<br>6.39	76.08<br>6.5	75.30<br>6.47	74.82<br>5.85	74.95<br>6.02	74.68<br>6.45	74.36<br>6.53
**Female*** (n,%)	60,709<br>66.84	57,617<br>64.73	50,203<br>62.52	46,425<br>60.40	8,718<br>46.42	7,603<br>44.88	5,400<br>42.45	4,230<br>39.09
**Race*** (n,%)								
White	81,708<br>89.96	79,729<br>89.60	71,298<br>88.81	67,461<br>87.77	17,220<br>91.70	15,309<br>90.43	11,404<br>89.68	9,545<br>88.23
Black	5,300<br>5.84	5,313<br>5.97	4,926<br>6.14	4,839<br>6.30	895<br>4.77	929<br>5.48	735<br>5.79	655<br>6.05
Hispanic	1,678<br>1.85	1,483<br>1.66	1,294<br>1.61	1,124<br>1.46	298<br>1.58	252<br>1.49	184<br>1.45	140<br>1.29
Asian	1,102<br>1.21	1,252<br>1.41	1,374<br>1.71	1,347<br>1.75	166<br>0.88	193<br>1.15	169<br>1.33	162<br>1.49
Other / Unknown	1,040<br>1.14	1,209<br>1.36	1,382<br>1.72	2,090<br>2.72	201<br>1.07	244<br>1.45	222<br>1.75	317<br>2.93
**Comorbid Conditions*** (n,%)								
Myocardial Infarction	4,726<br>5.35	4,674<br>5.32	4,044<br>5.15	3,858<br>5.13	1,907<br>10.45	1,396<br>8.31	999<br>8.07	945<br>8.95
Congestive Heart Failure	12,801<br>14.50	12,119<br>13.78	10,039<br>12.79	8,454<br>11.23	3,220<br>17.67	2,777<br>16.59	1,994<br>16.11	1,682<br>15.94
Peripheral Vascular Disease	11,849<br>13.41	13,476<br>15.37	13,285<br>16.93	12,242<br>16.25	3,066<br>16.83	3,229<br>19.46	2,663<br>21.51	2,417<br>22.90
Cerebrovascular Disease	12,404<br>14.04	13,395<br>15.27	12,646<br>16.11	11,078<br>14.72	3,183<br>17.47	3,099<br>18.61	2,435<br>19.64	2,086<br>19.76
Dementia	2082<br>2.36	2283<br>2.60	2065<br>2.63	1832<br>2.43	206<br>1.13	237<br>1.43	189<br>1.53	188<br>1.78
Chronic Pulmonary Disease	19,251<br>21.80	19,813<br>22.56	17,958<br>22.88	17,040<br>22.64	3,922<br>21.53	3,784<br>22.70	2,938<br>23.72	2,622<br>24.84
Rheumatic Disease	4145<br>4.69	4074<br>4.64	3777<br>4.81	3632<br>4.82	734<br>4.03	692<br>4.15	540<br>4.35	483<br>4.58
Peptic Ulcer Disease	2619<br>2.97	2035<br>2.31	1530<br>1.95	1320.2<br>1.75	532<br>2.92	377<br>2.25	240<br>1.93	192<br>1.82
Mild Liver Disease	2,702<br>3.06	3,404<br>3.88	3,605<br>4.60	3,953<br>5.25	501<br>2.75	546<br>3.29	517<br>4.18	522<br>4.95
Diabetes Without Complication	20,945<br>23.71	23,796<br>27.13	23,675<br>30.18	22,828<br>30.33	5,684<br>31.20	5,610<br>33.64	4,490<br>36.28	4,002<br>37.92
Diabetes With Complication	5,399<br>6.11	6,638<br>7.57	7,216<br>9.20	8,376<br>11.11	1,501<br>8.24	1,609<br>9.68	1,423<br>11.53	1,634<br>15.49
Hemiplegia / Paraplegia	979<br>1.11	843<br>0.96	694<br>0.88	632<br>0.84	166<br>0.91	140<br>0.84	103<br>0.83	94<br>0.89
Renal Disease	3,302<br>3.74	5,170<br>5.93	7,484<br>9.55	9,310<br>12.37	683<br>3.75	1,027<br>6.27	1,328<br>10.78	1,602<br>15.19
Malignancy	12,514<br>14.17	12,775<br>14.56	12,085<br>15.40	11,808<br>15.69	2,177<br>11.95	2,071<br>12.42	1,643<br>13.25	1,418<br>13.44
Moderate / Severe Liver Disease	169<br>0.19	206<br>0.23	209<br>0.27	252<br>0.33	23<br>0.12	29<br>0.17	22<br>0.17	27<br>0.26
Metastatic Solid Tumor	1,363<br>1.54	1,297<br>1.48	1,148<br>1.46	1,202<br>1.60	154<br>0.84	142<br>0.85	105<br>0.85	106<br>1.01
AIDS/HIV	30<br>0.03	35<br>0.04	50<br>0.06	65<br>0.09	9<br>0.05	11<br>0.06	11<br>0.09	12<br>0.11

* Counts and percentages are expressed per year, averaged over each time interval

© 2019 Kini V et al. *JAMA Network Open*.

eFigure 1. Annual Counts of Services per Beneficiary-Year Among the Overall Population (5% Medicare Fee-for-Service Sample) and the Study Cohorts Eligible for High- and Low-Value Testing

AMI hospitalizations (Panel A), HF hospitalizations (Panel B), low-risk surgeries (Panel C), and PCI/CABG procedures (Panel D)

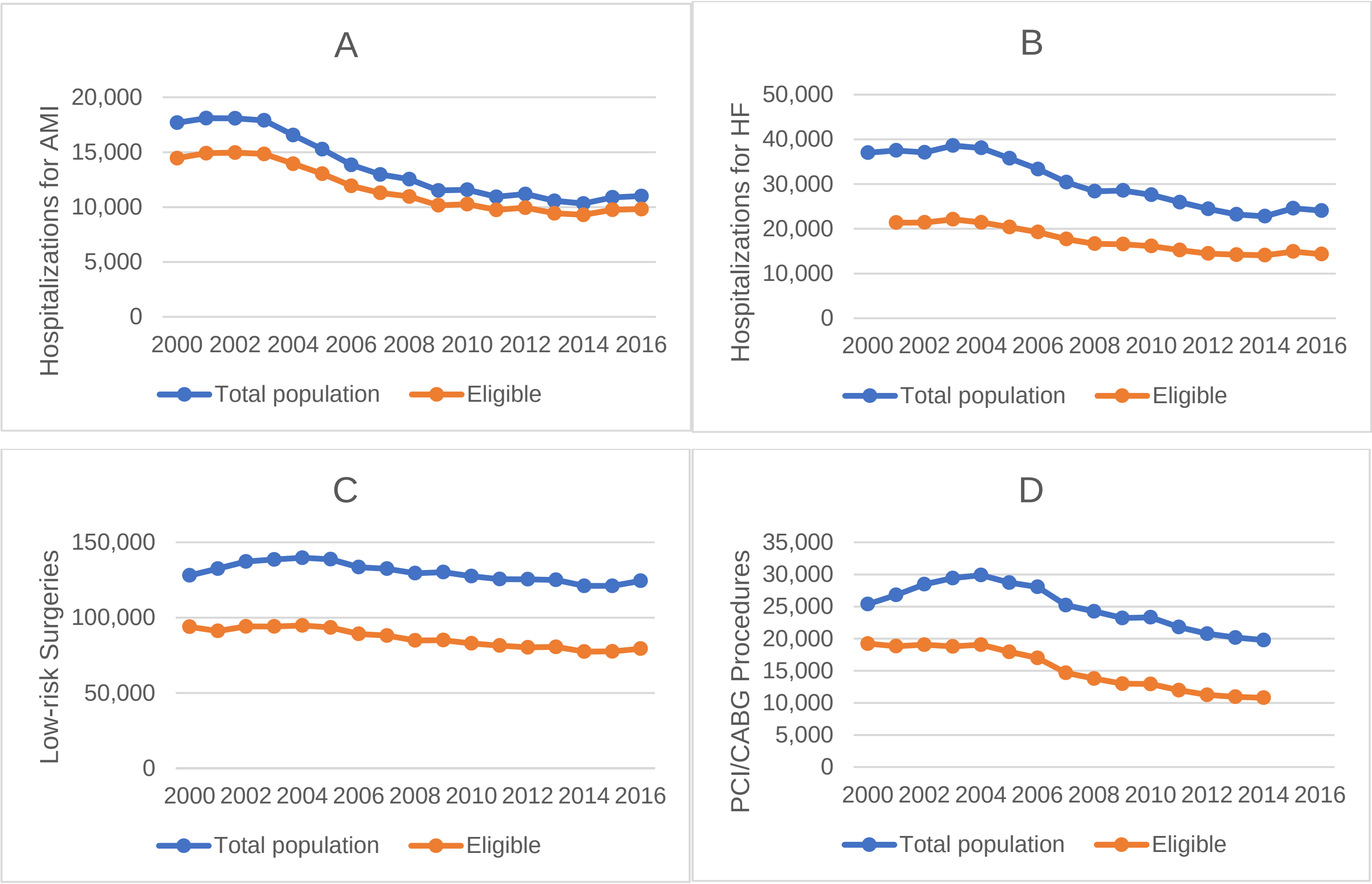

© 2019 Kini V et al. *JAMA Network Open*.

eFigure 2. AMI Sensitivity Analysis

Annual rate of high-value testing among AMI patients with and without ST-segment elevation (STEMI and non-STEMI)

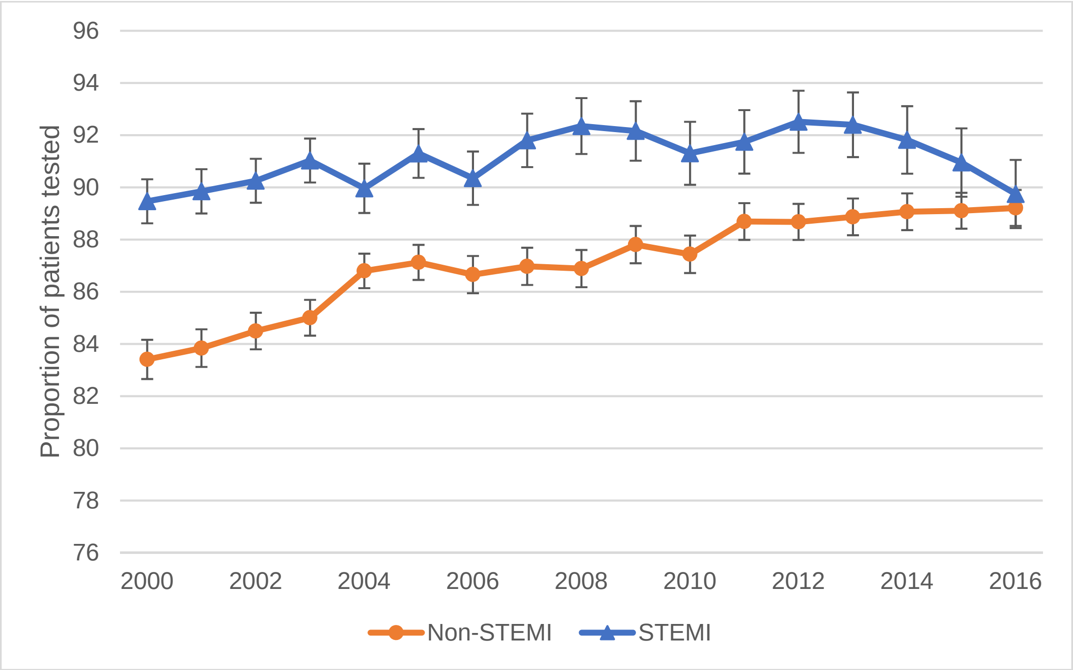

© 2019 Kini V et al. *JAMA Network Open*.

eFigure 3. Heart Failure Sensitivity Analysis: Annual Rate of High-Value Testing for Patients Hospitalized With Incident Heart Failure

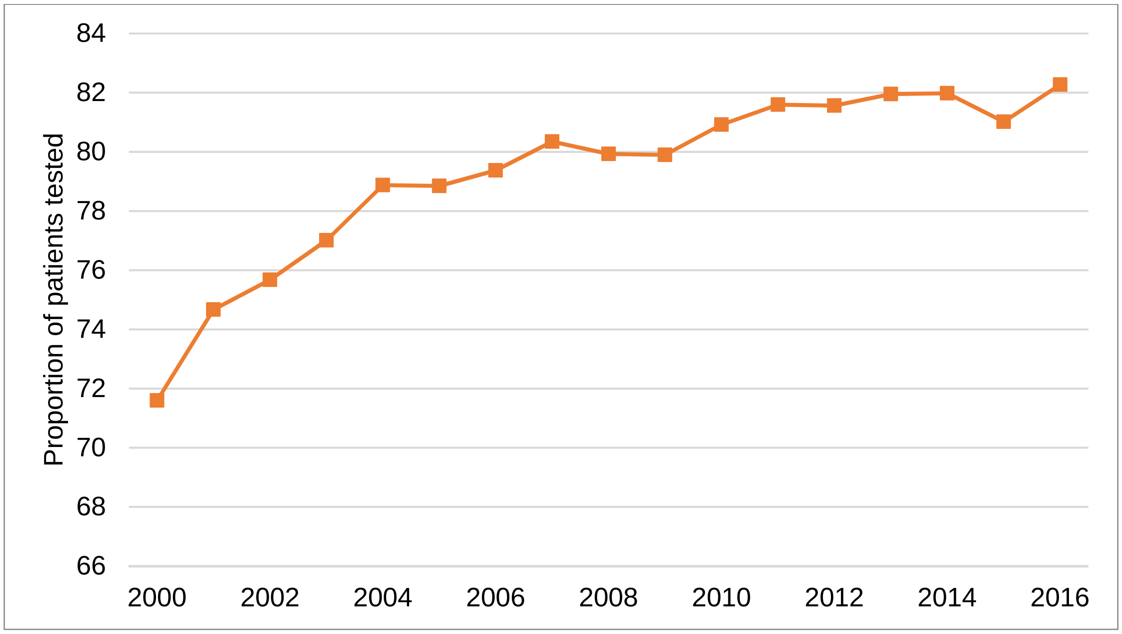

© 2019 Kini V et al. *JAMA Network Open*.

eFigure 4. Heart Failure Sensitivity Analysis: Trends in High-Value Testing Among HF Patients, Varying the Time Period for Measuring Testing Rates

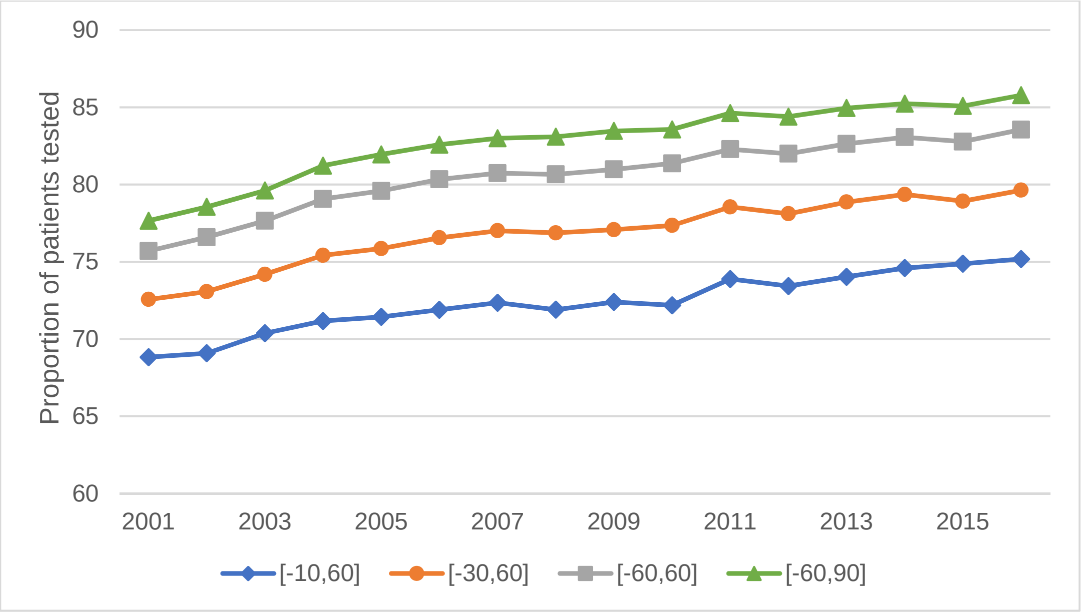

© 2019 Kini V et al. *JAMA Network Open*.

eFigure 5. Coronary Revascularization Sensitivity Analysis: Trends in Low-Value Testing Within 2 Years, 1 Year, and 6 Months of Coronary Revascularization

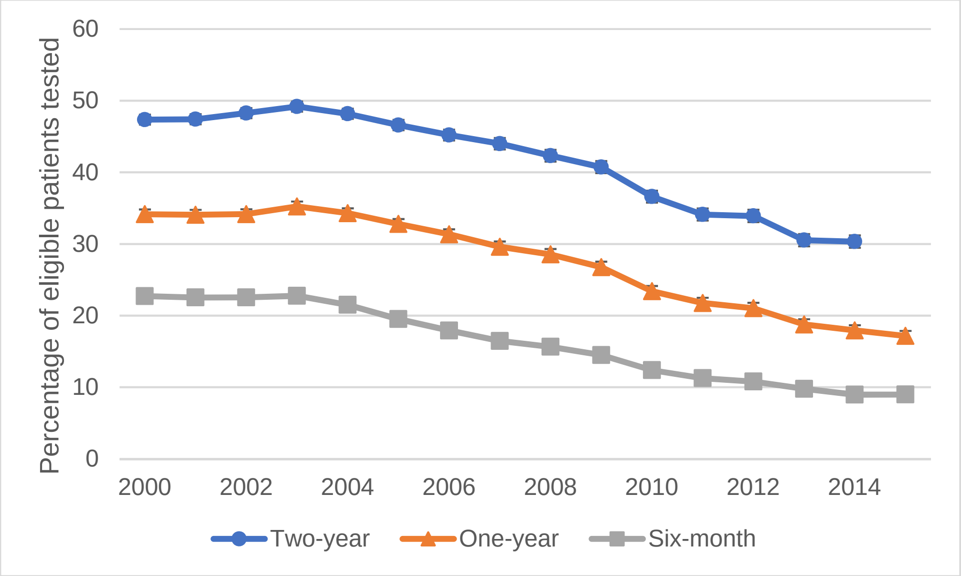

© 2019 Kini V et al. *JAMA Network Open*.

eFigure 6. Coronary Revascularization Sensitivity Analysis: Trends in Low-Value Testing Within 2 Years Without Excluding Tests That Were Followed by Subsequent PCI/CABG Within 30 Days

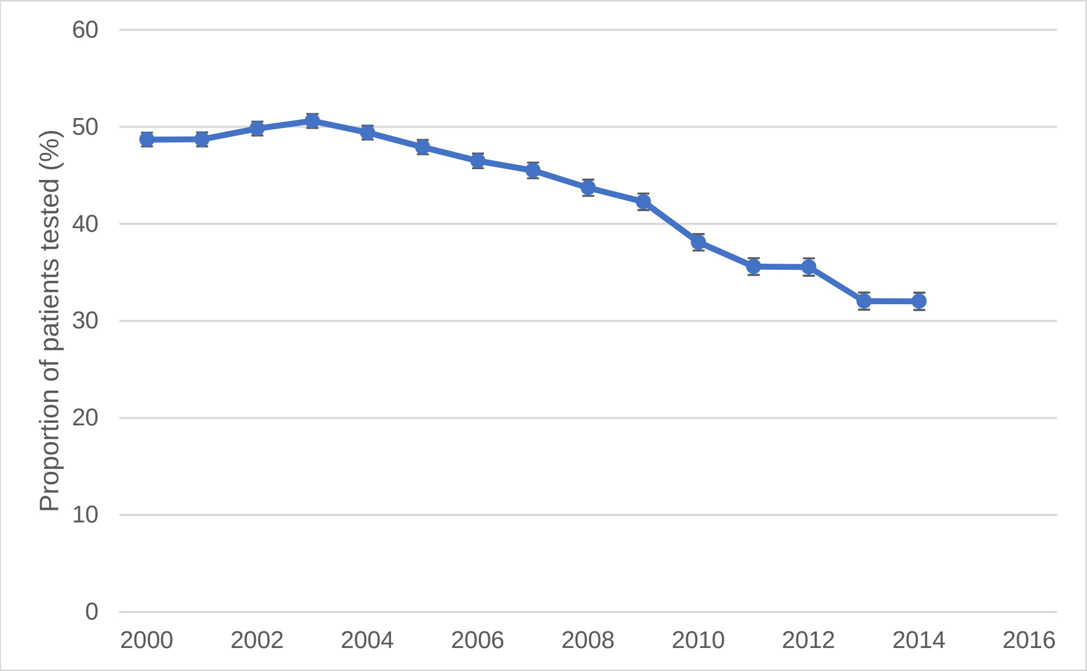

© 2019 Kini V et al. *JAMA Network Open.*

eFigure 7. Unadjusted Annual Rates of High- and Low-Value Testing

A-D: Unadjusted annual rates of high-value testing in the AMI cohort (Panel A) and HF cohort (Panel B), and low-value testing in the low-risk surgery cohort (Panel C) and coronary revascularization cohort (Panel D).

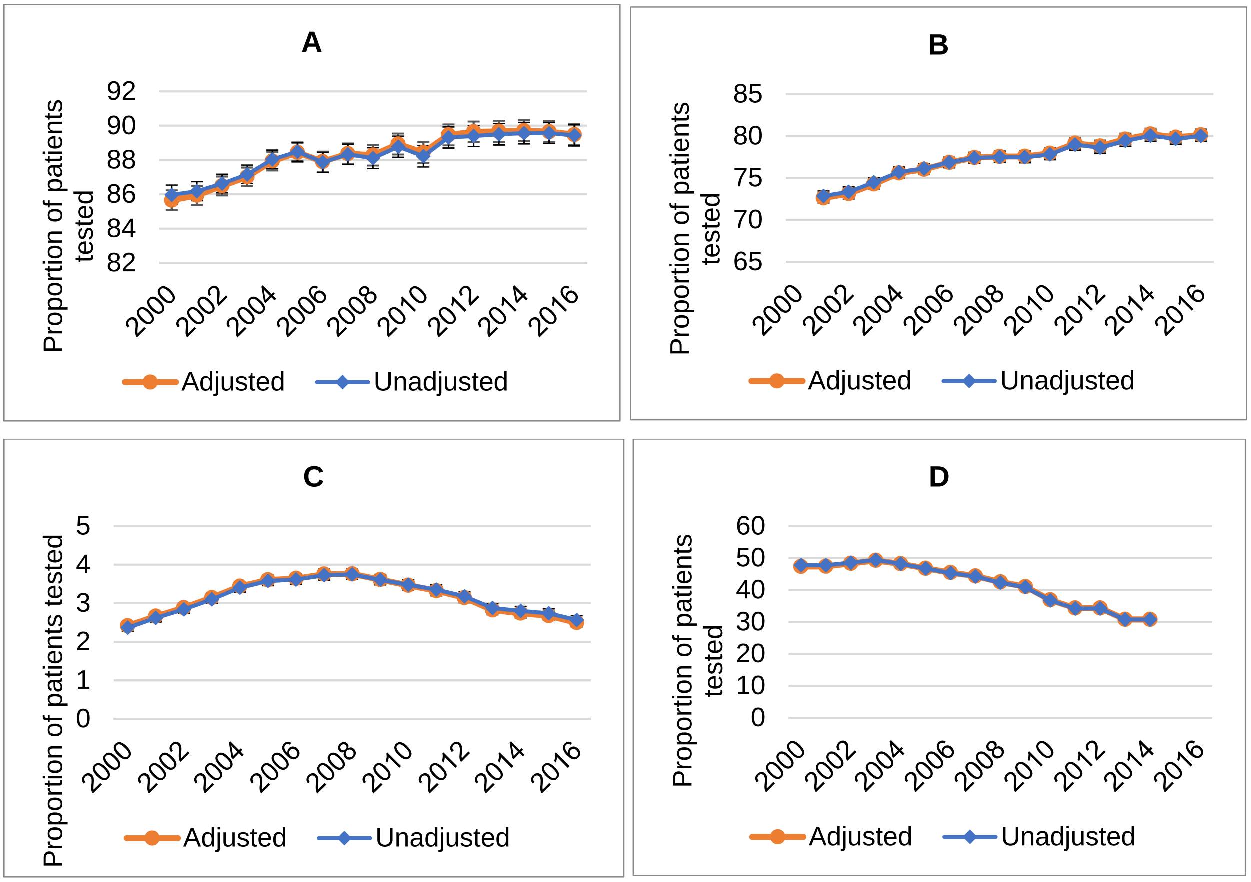

© 2019 Kini V et al. *JAMA Network Open.*
